# DOES DEMENTIA POLICY ACCOUNT FOR DIVERSITY? AN ANALYSIS OF CANADIAN DEMENTIA POLICIES

**DOI:** 10.1093/geroni/igz038.1601

**Published:** 2019-11-08

**Authors:** Kimberley Wilson

**Affiliations:** University of Guelph, Guelph, Ontario, Canada

## Abstract

Background: The World Report on Ageing and Health outlines key policy challenges that need to be addressed to have successful public health response to population aging, including ‘dealing with diversity’ and ‘reducing inequity.’ Dementia has been framed as a global health challenge affecting approximately 46 million people worldwide. To reduce inequality, dementia policies must account for and respond to diversity. The purpose of this research was to conduct an analysis of current Canadian federal, provincial, and territorial dementia strategies to examine their inclusion of dimensions of diversity. Method: We conducted an internet-based search and identified 13 unique Canadian federal, provincial, and territorial dementia documents. We completed a deductive content analysis to review each policy for content on: age and sex; racial and ethnic identity; sociocultural identity; religion; socioeconomic status; gender identity and sexual orientation; geographical location; and language fluency and communicative ability. Results: Within Canadian dementia policies there is minimal focus on diversity. When diverse identities were acknowledged in policies, very little guidance was provided to local policy-makers, healthcare administrators, or service providers in how to acknowledge and accommodate different group’ needs with services. Further, none of the policies adopted an intersectional approach; that is, they failed to recognize that older adults have several overlapping and interrelating identities. Conclusion: As Canada and other countries move towards developing and revising dementia policies it is imperative that they account for diversity within aging populations.

